# Selective Serotonin Reuptake Inhibitor-Treatment Does Not Show Beneficial Effects on Cognition or Amyloid Burden in Cognitively Impaired and Cognitively Normal Subjects

**DOI:** 10.3389/fnagi.2022.883256

**Published:** 2022-06-23

**Authors:** Yvonne Bouter, Caroline Bouter

**Affiliations:** ^1^Department of Psychiatry and Psychotherapy, University Medical Center Göttingen (UMG), Georg-August-University, Göttingen, Germany; ^2^Department of Nuclear Medicine, University Medical Center Göttingen (UMG), Georg-August-University, Göttingen, Germany

**Keywords:** positron emission tomography, selective serotonin reuptake inhibitors, amyloid-PET imaging, florbetaben, Alzheheimer’s disease

## Abstract

Preclinical studies indicate that selective serotonin reuptake inhibitors (SSRI) have beneficial effects on Alzheimer-related pathologies. Therefore, the aim of this study was to evaluate the influence of SSRI-treatment on amyloid burden in ^18^F-Florbetapir-positron emission tomography (PET) and on cognition in cognitively normal and cognitively impaired subjects. We included *n* = 755 cognitively impaired and *n* = 394 cognitively normal participants from the Alzheimer’s Disease Neuroimaging Initiative (ADNI) that underwent at least one ^18^F-Florbetapir-PET. Standardized uptake ratios (SUVR) and the Alzheimer Disease Assessment Scale-cognitive subscale (ADAS) scores as well as follow-up results were compared between subgroups with a history of SSRI-treatment (SSRI+) and without SSRI-treatment (SSRI-) as well as in subgroups of SSRI+/Depression+ and SSRI+/Depression- and SSRI-/Depression+ and SSRI-/Depression-. ^18^F-Florbetapir-PET did not show significant differences of SUVR between the SSRI+ and SSRI- groups in both, cognitively impaired and cognitively normal participants. There were no differences in subgroups of SSRI+/Depression+ and SSRI+/Depression- and SSRI-/Depression+ and SSRI-/Depression-. However, SUVR showed a dose-dependent inverse correlation to the duration of medication in cognitively normal and in cognitively impaired patients. SRRI-treatment did not show an effect on ADAS scores. Furthermore, there was no effect on follow-up SUVR or on follow-up ADAS scores. Overall, SSRI-treatment did not show beneficial effects on amyloid load nor on cognition.

## Introduction

Alzheimer’s disease (AD), the most common form of dementia, is a progressive neurogenerative disease characterized by memory loss and decline of cognitive function. Despite extensive treatment efforts, AD remains incurable and novel therapies to prevent, slow down or delay the onset of the disease are urgently needed. Without effective therapies, the number of patients with dementia worldwide is estimated to reach more than 130 million by 2050 (Cummings et al., 2016).

Given the need for disease-modifying therapies for AD, drug repurposing may be a promising approach. Preclinical studies indicate that antidepressants, particularly selective serotonin reuptake inhibitors (SSRI), have beneficial effects on AD-related biomarkers including amyloid plaques, one of the major pathological hallmarks in AD (Cirrito et al., 2011, 2020; Sheline et al., 2014). Neuritic plaques consist of aggregated Abeta peptides that are formed within neurons by sequentially cleavage of the amyloid precursor protein (APP). Dysregulation of Aβ production and Aβ clearance leads to accumulation of hydrophobic Aβ forms and the formation of extracellular plaques (Chen et al., 2017; Guo et al., 2020). While the pathway of APP processing is well-characterized, mechanisms of its regulation are not yet fully understood. APP processing can be modulated by several signaling pathways including NMDA, acetylcholine and serotonin signaling systems. Serotonin receptors (5-HTr) might affect APP processing and Aβ levels. *In vitro* studies could show that activation of 5-HTr2a, 5-HTr2c, and 5-HTr4 increases non-amylogenic APP processing (Nitsch et al., 1996; Robert et al., 2001; Pakaski et al., 2005; Shen et al., 2011). In addition, treatment with SSRI reduced Aβ levels and amyloid plaque burden in different mouse models of AD (Tucker et al., 2005; Nelson et al., 2007; Cirrito et al., 2011, 2020). Furthermore, beneficial effects of antidepressant medication on cognition could be shown in depressed patients, while there was no effect on non-depressed individuals (Prado et al., 2018).

However, whether chronic SSRI-treatment influences amyloid burden and cognition in humans remains unclear. A few studies suggest a possible positive benefit of SSRI treatment on the risk of developing AD, whereas the effect on amyloid burden remains controversial (Kessing et al., 2009; Cirrito et al., 2011; Bartels et al., 2018, 2020).

The aim of this study is the evaluation of the influence of SSRI-treatment on amyloid burden in ^18^F-Florbetapir positron emission tomography (PET) in cognitively normal and cognitively impaired individuals.

## Materials and Methods

Data used in this study were obtained from the Alzheimer’s Disease Neuroimaging Initiative (ADNI) database.^1^ The ADNI was launched in 2003 as a public-private partnership, led by Principal Investigator Michael W. Weiner, MD. The primary goal of ADNI has been to test whether serial magnetic resonance imaging (MRI), positron emission tomography (PET), other biological markers, and clinical and neuropsychological assessment can be combined to measure the progression of mild cognitive impairment (MCI) and early Alzheimer’s disease (AD). ADNI is a multicentered project that aims to improve clinical research on AD unifying data on demographics, clinical and cognitive assessments as well as on genetic, biochemical and imaging biomarkers. Standardized protocols and unlimited data access allow analysis of an enormous amount of data from more than 1800 patients that have been included to the ADNI database to date. Groups of AD patients, patients with mild cognitive impairment (MCI), and cognitively normal elderly controls were included in four phases starting in 2004.

### Study Sample

**FIGURE 1:**
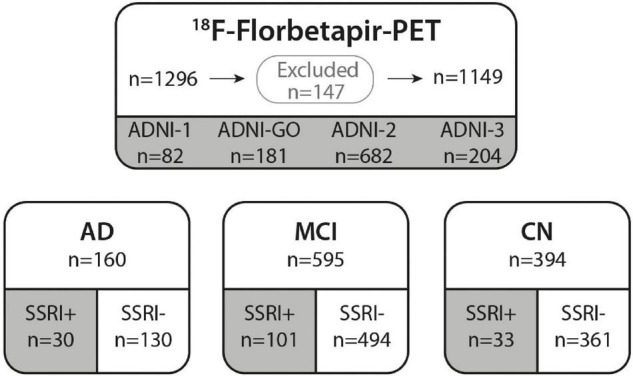
ADNI cohort. ^18^F-Florbetapir-PET was performed in *n* = 1296 ADNI participants. *N* = 147 subjects were excluded from our study due to missing data or stopped SSRI medication as well as patients with subjective memory deficits that did not meet criteria of AD or MCI. A total of *n* = 1149 subjects from ADNI phases 1, GO, 2 and 3 were included in this study. All subjects were categorized according to their diagnosis in AD, MCI, or CN. Groups were further subdivided in subjects with prior SSRI-treatment (SSRI+) and subjects with no history of SSRI-treatment (SSRI−). AD, Alzheimer’s dementia; MCI, mild cognitive impairment; CN, cognitively normal controls.

In this study, ADNI data from all four phases, ADNI-1, ADNI-GO, ADNI-2, and ADNI-3, were downloaded from the ADNI database (see text footnote 1) on May 7, 2021. Patients aged 57–93 with at least one available ^18^F-Florbetapir-PET were downloaded (*n* = 1296; Figure 1). Patient characteristics, medical history, medication, Mini Mental State Examination (MMSE), Clinical Dementia Rating score (CDR), the Alzheimer’s Disease Assessment Scale–Cognitive Subscale (ADAS), depression-history, education years, ApoE4 status and PET results were assessed. Patients with insufficient or inconsistent data on cognition or prior medication were excluded (*n* = 22). *N* = 20 patients were excluded as SSRI-treatment was paused before the baseline PET and *n* = 105 patients without significant objective amnestic dysfunction but with subjective memory concerns were excluded.

According to the ADNI protocol, participants were categorized as cognitively normal and cognitively impaired with subgroups of MCI and Alzheimer’s dementia (AD). AD patients showed a significant subjective and objective memory loss (based on scores on the WMS-R Logical Memory II subscale) affecting activities of daily living with a MMSE below 26 and CDR of 0.5 or 1 plus. MCI patients were defined as patients showing significant memory issues with a MMSE from 24 to 30 and CDR of 0.5 plus an abnormal score on the WMS-R Logical Memory II subscale. Cognitively normal participants showed no signs of dementia with normal cognition (MMSE from 24 to 30 and a CDR of 0 plus a WMS-R Logical Memory II subscale score above education-adjusted cutoffs).

Detailed information about the ADNI inclusion and exclusion criteria can be found online.^2^

For our analysis, all groups and subgroups were divided into history of SSRI treatment (SSRI+) and without SSRI treatment (SSRI−).

A total of *n* = 164 patients were currently treated with an SSRI at the time of the PET (Citalopram: *n* = 69; Escitalopram *n* = 16; Fluoxetine *n* = 24; Paroxetine *n* = 7; Sertraline *n* = 48; Table 1).

**TABLE 1:** Selective serotonin reuptake inhibitor (SSRI) treatment.

	SSRI+		Duration of medication	Dose
	*n*	%	Months (SD)	mg (SD)
All	164	100	56.27 (64.92)	
Citalopram	69	42	43.75 (50.61)	19.64 (10.79)
Escitalopram	16	10	47.27 (47.41)	14.06 (6.88)
Fluoxetine	24	15	113.5 (94.96)	28.33 (14.94)
Paroxetine	7	4	39.0 (28.65)	25.71 (12.72)
Sertraline	48	29	54.23 (63.08)	72.86 (43.71)

### Alzheimer’s Disease Neuroimaging Initiative ^18^F-Florbetapir-PET/CT

^18^F-Florbetapir-PET/CT was performed according to a standardized imaging protocol with 4 × 5min frames acquired 50–70 min post-injection of 370 (± 10%) MBq ^18^F-Florbetapir. All PET images were reviewed for quality control by the ADNI PET QC team and transmitted in DICOM format to the Laboratory of Neuroimaging (LONI) for storage. In order to uniformize data from different origins, PET image data were pre-processed with motion correction, time frame averaging, reorientation in a standardized 160 × 160 × 96 matrix with a voxel size of 1.5 mm and smoothing with a scanner-specific filter function determined from Hoffman phantom scans during the certification process (more details on adni.loni.usc.edu).

PET images were further processed with FreeSurfer v7.1.1 for an MRI-based definition of multiple cortical regions as well as reference regions for normalization.

Amyloid load was quantitatively analyzed using standardized uptake ratios (SUVR) of composite regions normalized by cerebellar uptake. In order to analyze possible region-specific differences, SUVR of frontal, parietal, temporal and cingulate cortex regions were also obtained. As described before, intensity normalization of SUVRs was performed using a FreeSurfer-defined whole cerebellum region for cross-sectional analysis and a FreeSurfer-defined composite reference region for longitudinal studies.

### Statistical Analysis

Statistical analysis was performed using SPSS Statistics version 27 (IBM, Armonk, NY, United States) and GraphPad Prism version 9 (GraphPad Software, San Diego, CA, United States).

Differences between groups were tested using non-parametric tests (as data did not pass normality tests, e.g., Shapiro–Wilk test *p* = 0.0006), therefore Mann–Whitney *U* test or Kruskal–Wallis test followed by *post hoc* multiple comparison were used as indicated. Univariate analysis of covariance (ANCOVA) was used for covariate adjustment as indicated. Chi-square test or Fisher’s exact test were used for categorical variables. Relationships between two variables were assessed using Spearman correlation and simple linear regression.

For follow-up PET and ADAS data changes were calculated as absolute and relative change between baseline and follow-up (ΔSUVR/ΔADAS; Δ%SUVR/Δ%ADAS).

Significance levels are given as follows: *p < 0.05; ^**^p < 0.01; ^***^p < 0.001.

## Results

### Baseline Characteristics

A total of *n* = 755 cognitively impaired (AD and MCI patients) and *n* = 394 cognitively normal participants from ADNI phases ADNI-1 (*n* = 82), ADNI-GO (*n* = 181), ADNI-2 (*n* = 682), and ADNI-3 (*n* = 204) that underwent at least one ^18^F-Florbetapir-PET were included in this study. Baseline characteristics for all patients categorized into diagnostic groups are shown in Table 2. Patients were categorized as AD (*n* = 160), MCI (*n* = 595) and cognitively normal (*n* = 394) according to clinical symptoms and results of neuropsychological assessments following the ADNI protocol (Figure 1).

**TABLE 2:** Patient characteristics.

Characteristics	Total		SSRI+		SSRI−		
**1. Cognitively impaired patients**							

	**Total *N* = 755**		**SSRI+ *N* = 131**		**SSRI− *N* = 624**		
	***Mean***	***SD***	***Mean***	***SD***	***Mean***	***SD***	***p***

Age	73.59	7.9	73.04	7.6	73.7	8.0	0.4095
MMSE	25.96	3.9	25.52	4.01	26.1	3.8	0.2549
ADAS	19.66	11.2	22.43	12.1	19.0	10.8	0.0126
Education (years)	16	2.7	15.73	2.6	16.06	2.7	0.185

	***N***	***%***	***N***	***%***	***N***	***%***	

*Diagnosis*							>0.9999
AD	160	21	30	21	130	21	
MCI	595	79	101	79	494	79	
*Gender*							0.1146
Female	326	44	65	49	261	42	
Male	429	56	66	51	363	58	
*ApoE4 alleles*							0.2385
0	378	50	56	43	322	52	
1	287	38	57	43	230	37	
2	90	12	18	14	72	12	
*SSRIs*							
All			131	100			
Citalopram			52	40			
Escitalopram			9	7			
Fluoxetine			22	16			
Paroxetine			5	4			
Sertraline			39	32			
*History of depression*							<0.001
Depression	249	33	94	69	155		
No depression	506	67	37	31	469		

**2. Objectively cognitively normal patients**							

	**Total *N* = 394**		**SSRI+ *N* = 33**		**SSRI− *N* = 361**		
	***Mean***	***SD***	***Mean***	***SD***	***Mean***	***SD***	***p***

Age	74.44	7.13	72.08	5.8	74.2	6.9	0.1930
MMSE	28.99	1.21	28.68	1.7	29.0	1.2	0.3556
ADAS	9.3	5.0	11.5	7.8	9.1	4.5	0.0383
Education (years)	16.54	2.56	15.64	2.25	16.66	2.57	0.0138

	***N***	***%***	***N***	***%***	***N***	***%***	

*Gender*							0.0035
Female	215		26	79	189	52	
Male	179		7	21	172	48	
*ApoE4 alleles*							0.5615
0	282		24	73	258	72	
1	100		9	27	91	25	
2	12		–	0	12	3	
*SSRI*							
All			33	100			
Citalopram			17	52			
Escitalopram			7	21			
Fluoxetine			2	6			
Paroxetine			2	6			
Sertraline			5	15			
*History of depression*							<0.001
Depression	51	13	19	58	32	9	
No depression	343	87	14	42	329	91	

*AD, Alzheimer’s dementia; MCI, mild cognitive impairment. Gray shades highlight patients with SSRI medication.*

The group of AD patients was slightly older compared to the MCI group (Kruskal–Wallis test with Dunn’s multiple comparison test, *p* = 0.0093) while there were no differences between all other groups (Kruskal–Wallis test, *p* > 0.07). Groups of cognitively impaired patients showed a higher number of male patients compared to cognitively normal controls (Chi-square test, *p* = 0.0002).

MMSE, CDR and ADAS showed significant differences between AD and MCI as well as between AD or MCI compared to cognitively normal patients after adjusting for age and gender as covariates (ANCOVA, *p* < 0.001, Table 2).

There were no differences between age, gender, MMSE scores, ApoE4-allels or diagnosis between cognitively impaired SSRI+ and SSRI− patients (*p* > 0.11; Table 2). There were no differences between age, MMSE scores or ApoE4-allels between cognitively normal SSRI+ and SSRI− patients (*p* > 0.19; Table 2). Slight differences were observed in gender distribution with proportionately more women in the SSRI+ group (Chi-square test; *p* = 0.002; Table 2) which was considered irrelevant for further analysis. Amyloid load did not differ between female and male patients (Mann–Whitney *U* test, *p* = 0.1308).

*N* = 300 patients had a history of depression. The amount of patients with a history of depression was significantly higher in the SSRI+ group compared to the SSRI− group (Chi-square test; *p* < 0.001; Table 2).

### Positron Emission Tomography Results

**FIGURE 2:**
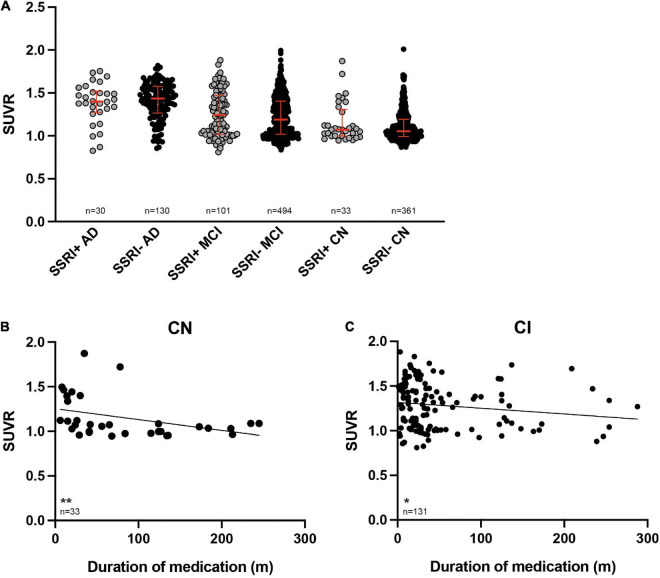
^18^F-Florbetapir uptake in SSRI-treated and untreated subjects. **(A)** SUVR was significantly higher in AD-patients compared to MCI patients as well as between AD or MCI patients compared to CN (ANCOVA). SUVR did not show significant differences between SSRI-treated (SSRI+) and untreated (SSRI−) subjects regardless of the diagnosis (Mann–Whitney *U* test; red bars represent median ± interquartile range). **(B)** SUVR showed a time-dependent inverse correlation to the duration of medication in cognitively normal patients. **(C)** SUVR also showed a time-dependent negative relation to the duration of medication in cognitively impaired patients. Spearman correlation; *p < 0.05; **p < 0.01. SUVR, standard uptake value ratio; CN, cognitively normal controls; CI, cognitively impaired patients; AD, Alzheimer’s disease; MCI, mild cognitive impairment.

A baseline PET was available in all patients (*n* = 1149). SUVRs were significantly higher in the AD subgroup compared to the MCI subgroup as well as in AD or MCI compared to cognitively normal controls after adjusting for age and gender as covariates (Figure 2A, ANCOVA, *p* < 0.001).

### Selective Serotonin Reuptake Inhibitors Treatment

There were no significant differences of SUVR between the SSRI+ and SSRI− patients regardless of the diagnosis (Figure 2A, Mann–Whitney *U* test; AD: *p* = 0.667; MCI: *p* = 0.169; cognitively normal controls: *p* = 0.188).

SUVR showed a significant negative correlation with the duration of SSRI-treatment in cognitively normal SSRI+ as well as in cognitively impaired SSRI+ patients (Figures 2B,C, Spearman correlation, cognitive normal: *p* = 0.0038; *r* = −0.4965; cognitively impaired: *p* = 0.025; *r* = −0.1958).

SUVRs did not differ between groups treated with citalopram, escitalopram, fluoxetine, paroxetine or sertraline, neither in SSRI+ CI patients (Kruskal–Wallis test; *p* = 0.3276) nor in SSRI+ cognitive normal controls (Kruskal–Wallis test; *p* = 0.8296).

There was no dose-dependency of SUVR in any of the groups treated with citalopram, escitalopram, fluoxetine, paroxetine or sertraline, neither in SSRI+ cognitively impaired patients (Kruskal–Wallis test; *p* > 0.14) nor in SSRI+ cognitive normal patients (Kruskal–Wallis test; *p* > 0.3).

### Regional Differences

Regional differences of amyloid load were analyzed in frontal, cingulate, parietal and temporal cortex. There were no significant differences between regional SUVR of SSRI+ and SSRI− regardless of the diagnosis (Kruskal–Wallis test, *p* > 0.5 in all groups).

### History of Depression

**FIGURE 3:**
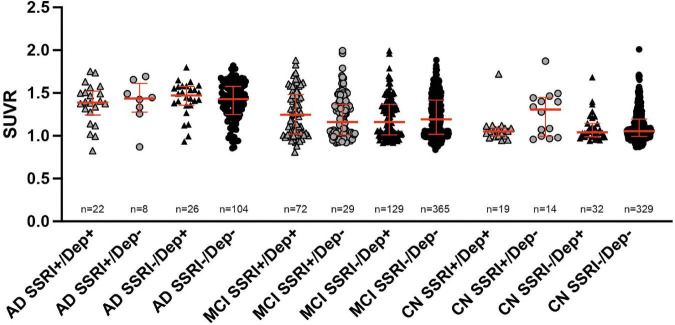
^18^F-Florbetapir uptake in SSRI-treated and untreated subjects with and without history of depression. No significant differences between SSRI-treated and untreated subjects with or without depression were detected regardless of the diagnosis (Kruskal–Wallis test; red bars represent median ± interquartile range. AD, Alzheimer’s disease; MCI, mild cognitive impairment; CN, cognitively normal controls.

^18^F-Florbetapir uptake did not show significant differences between SSRI+ and SSRI− patients with and without history of depression regardless of the diagnosis (Figure 3; Kruskal–Wallis test; AD: *p* = 0.8304; MCI: *p* = 0.5308; cognitive normal controls: *p* = 0.1937).

### Follow-Up ^18^F-Florbetapir-PET

At least one follow-up ^18^F- Florbetapir-PET was available in *n* = 650 patients. Mean follow-up period was 26.49 months for one follow-up PET. A second or third follow-up was only available in the MCI and cognitively normal group with a mean period of 57.34 months for a second follow-up PET scan (*n* = 361) and 74.86 months for a third follow-up PET scan (*n* = 184).

**FIGURE 4:**
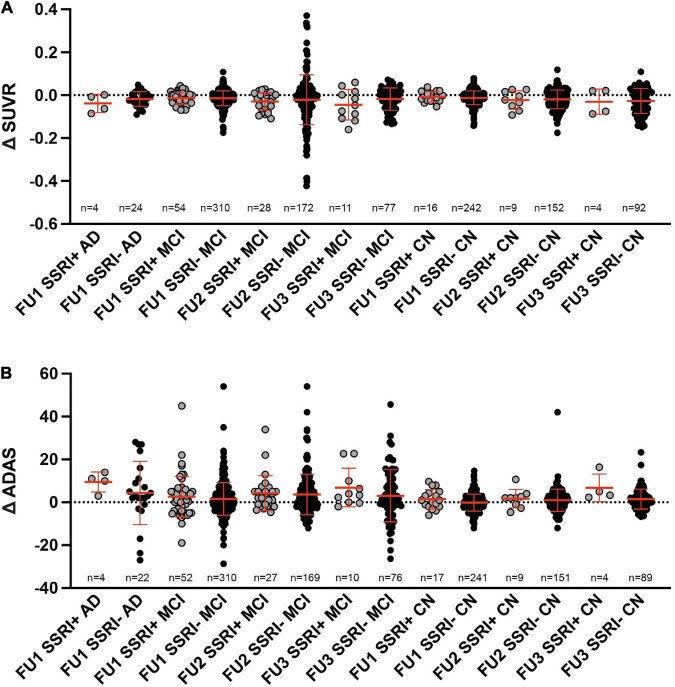
Follow up. **(A)** Differences of SUVR between follow-up and baseline PET did not show significant differences between SSRI-treated and untreated subjects (Mann–Whitney *U* test). **(B)** Differences of ADAS between follow-up and baseline examination did not show significant differences between SSRI-treated and untreated subjects (Mann–Whitney *U* test). Red bars represent median ± interquartile range.

In order to analyze changes of amyloid deposition, differences of SUVR between the baseline PET and available follow-up PETs were calculated (ΔSUVR). Longitudinal changes of SUVR did not show any significant differences between SSRI+ and SSRI− AD-patients after one follow-up (Table 3 and Figure 4A; Mann–Whitney *U* test; *p* = 0.4269).

**TABLE 3:** Follow up SUVR and ADAS.

	Delta SUVR			Delta ADAS		
	FU1	FU2	FU3	FU1	FU2	FU3
SSRI+ AD	+0.039 (4%)	na	na	+9.5 (29%)	na	na
SSRI− AD	+0.018 (1.8%)	na	na	+4.4 (14%)	na	na
SSRI+ MCI	+0.014 (1.6%)	+0.029 (3.3%)	+0.045 (4.9%)	+2.6 (14%)	+4.3 (22%)	+6.9 (36%)
SSRI− MCI	+0.014 (1.6%)	+0.021 (2.5%)	+0.018 (2.1%)	+1.6 (10%)	+3.7 (23%)	+3.1 (19%)
SSRI+ CN	+0.007 (1%)	+0.023 (3%)	+0.03 (3.8%)	+1.4 (11%)	+1.7 (13%)	+6.7 (46%)
SSRI− CN	+0.013 (1.7%)	+0.019 (2.5%)	+0.02 (2.6%)	−0.1 (1.1%)	+0.9 (10%)	+1.5 (17%)

*AD, Alzheimer’s dementia; MCI, mild cognitive impairment; FU, follow up; CN, cognitively normal controls. Gray shades highlight patients with SSRI medication.*

There were no significant differences between SSRI+ and SSRI− MCI-patients after the first, second or third follow-up (Table 3 and Figure 4A; Mann–Whitney *U* test; first follow-up: *p* = 0.9501; second follow up: *p* = 0.3405; third follow-up: *p* = 0.2387). Furthermore, we did not detect significant differences between ΔSUVR in the cognitively normal group either (Table 3 and Figure 4A; Mann–Whitney *U* test; first follow-up: *p* = 0.6367; second follow up: *p* = 0.8637; third follow-up: *p* = 0.921).

### Cognition

In order to analyze differences in cognition as well as longitudinal changes of cognition between SSRI+ and SSRI− groups the Alzheimer Disease Assessment Scale-cognitive subscale (ADAS) scores were used. Baseline ADAS as well as follow-up scores were collected as close to the date of the corresponding baseline and follow-up PET scan as possible.

**FIGURE 5:**
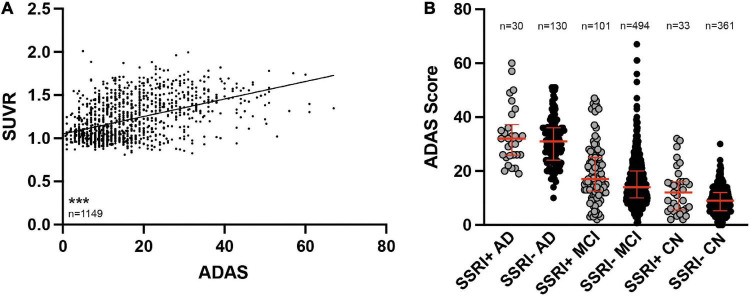
ADAS cognition test results. **(A)** ADAS scores correlated to SUVR results (Spearman correlation; ***p < 0.001). **(B)** SSRI-treatment did not influence cognition. ADAS was significantly higher in AD patients compared to MCI and both, AD patients and MCI patients showed higher ADAS scores compared to CN (ANCOVA). Red bars represent median with interquartile range. AD, Alzheimer’s disease; MCI, mild cognitive impairment; CN, cognitively normal controls.

Baseline ADAS was available in all patients (*n* = 1149). ADAS scores correlated with amyloid burden in PET (Figure 5A, Spearman *r* = 0.4396; *p* < 0.001). ADAS was significantly higher in AD patients compared to MCI patients and in MCI and AD patients compared to cognitively normal controls after adjusting for age, gender and education as covariates (Figure 5B; ANCOVA; *p* < 0.001). There were no significant differences between SSRI+ and SSRI− subgroups after adjusting for age, gender and education as covariates (Figure 5B, ANCOVA, AD: *p* = 0.238; MCI: *p* = 0.153; NMC: *p* = 0.063).

At least one follow-up ADAS score was available in *n* = 646 patients with a mean follow-up of 25.5 months. A second and third follow-up ADAS score was available in the MCI and cognitively normal group with a mean period of 60.67 months for a second follow-up (*n* = 356) and 73.93 months for a third follow-up (*n* = 179). In order to analyze longitudinal changes of cognition, differences of ADAS between the baseline score and available follow-up scores were calculated (ΔADAS; Table 3).

There were no significant differences of the ADAS score between SSRI+ and SSRI− AD-patients after one follow-up (Table 3 and Figure 4B; Mann–Whitney *U* test; *p* = 0.2593). Furthermore, we did not detect significant differences between ΔADAS in the MCI-group nor in the cognitively normal group after the first, second or third follow-up (Table 3 and Figure 4B; Mann–Whitney *U* test; MCI: first follow-up: *p* = 0.5651; second follow up: *p* = 0.5142; third follow-up: *p* = 0.2465; cognitively normal group: first follow-up: *p* = 0.2084; second follow up: *p* = 0.595; third follow-up: *p* = 0.359).

#### ApoE4

**FIGURE 6:**
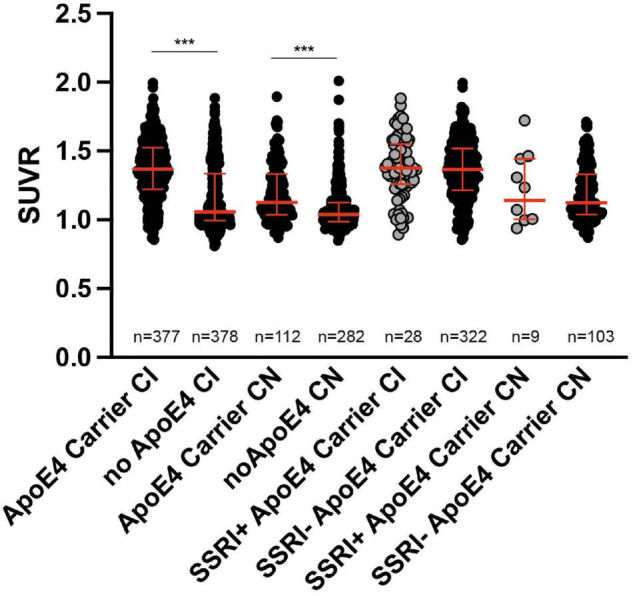
ApoE4 carriers. ApoE4 carriers showed significantly higher SUVR compared to non-carriers in cognitively impaired patients as well as in cognitively normal subjects (Mann–Whitney *U* test; ***p < 0.001). There were no differences between ApoE4 carriers in SSRI+ and SSRI− groups (Mann–Whitney *U* test). Red bars represent median ± interquartile range.

ApoE4 carriers showed significantly higher SUVR compared to non-carriers in cognitively impaired patients as well as in cognitively normal subjects (Mann–Whitney *U* test; *p* < 0.001; Figure 6). However, there were no differences in the SUVR between SSRI+ and SSRI− ApoE4 carriers regardless of the diagnosis (Mann–Whitney *U* test; AD: *p* = 0.7909; MCI: *p* = 0.3644; cognitively normal: *p* = 0.9065; Figure 6).

## Discussion

Amyloid plaques are a major pathological hallmark of AD and the modulation of plaques, APP processing, and related signaling pathways has been a research focus for novel AD-therapies for many years. The role of different neurotransmitter systems in the development of AD is still unclear. Recent evidence suggests that the serotonergic system, that has mainly been studied in mood disorders so far, might play a role in the pathogenesis of AD (Cirrito et al., 2011; Ramos-Rodriguez et al., 2013).

Mid-life or late-life depression is considered a risk factor for the development of AD. However, whether depression is a prodromal symptom or a true etiologic risk factor for AD remains controversial. Both, AD and depression share similar neuropathological changes as neuroinflammation, elevated oxidative stress markers and synaptic dysfunctions and an increased cortical amyloid burden is also seen in patients with a lifetime history of depression (Chung et al., 2015; Herbert and Lucassen, 2016; Mahgoub and Alexopoulos, 2016).

Therefore, the modulation of serotonergic systems with SSRIs might be a potential treatment option for AD patients with or without a history of depression.

Several preclinical studies demonstrated beneficial effects of SSRI-treatment on AD-pathologies in different mouse models of AD suggesting a modulation of APP processing by SSRIs. Cirrito et al. (2011) first showed a serotonin-dependent reduction of Aβ levels in the brain interstitial fluid (ISF) in APP/PS1 mice after short-term treatment with citalopram or fluoxetine. Furthermore, reduced cortical and hippocampal plaque burden as well as decreased Aβ levels in ISF and cerebrospinal fluid (CSF) were also detected after chronic citalopram administration (Cirrito et al., 2011). Beneficial effects on plaque load and Aβ-levels following citalopram treatment could also be confirmed by a couple of other studies (Sheline et al., 2014; Wei et al., 2017; Zhang et al., 2018). Sheline and colleagues demonstrated a reduced formation of plaques as well as decreased growth of preexisting plaques in APP/PS1 mice after 28 days of citalopram treatment (Sheline et al., 2014). Similar to the results obtained after citalopram treatment, several studies demonstrated that fluoxetine treatment decreases amyloid plaques and soluble Aβ levels in the brain in different AD mouse models (Cirrito et al., 2011; Wang et al., 2014, 2016; Qiao et al., 2016; Jin et al., 2017; Ma et al., 2017; Sun et al., 2017; Zhou et al., 2019). Furthermore, there is limited evidence that paroxetine has also beneficial effects on the AD pathology as Nelson et al. (2007) showed reduced accumulation of tau-protein and cortical Aβ1-40 levels after chronic paroxetine treatment in 3xTg mice and Olesen et al. (2017) detected a reduction of plaque load in the hippocampus of APP/PS1-mice (Nelson et al., 2007; Olesen et al., 2016, 2017). In addition, escpitalopram was able to reduce tau-hyperphosphorylation in cultures of Aβ1-42 treated hippocampal neurons of fetal brains obtained from rats (Wang et al., 2016) and in a recent study, Cirrito and colleagues showed a significantly reduced plaque load in APP/PS1 mice after 28 days of escpitalopram treatment (Cirrito et al., 2020).

While these preclinical results hint to a beneficial effect of SSRIs on AD-pathologies, studies on their impact in humans are very limited. Bartels et al. (2018) showed that long-term use of SSRIs delayed the conversion from MCI to AD in patients with a previous depression for 3 years (Bartels et al., 2018). Similarly, Burke et al. (2018) showed that the risk of cognitively normal patients with a prior history of depression developing AD was neutralized by SSRI treatment compared to untreated patients (Burke et al., 2018). A retrospective study of Down syndrome patients with a previous history of depression showed that SSRI use for more than 90 days significantly delayed the onset of dementia (Tsiouris et al., 2014).

Sheline et al. (2014) demonstrated a significant reduction of Aβ production and CSF Aβ concentration after a single high dose application of citalopram in healthy volunteers (Sheline et al., 2014). However, it should be noted that the study was performed on young participants, presumably before plaque formation while our cohort was much older. However, in a recent study, the same group showed that short-term longitudinal treatment of escitalopram decreases CSF Aβ42 levels in cognitively normal older adults (Sheline et al., 2020).

In the current study, we could not detect any differences of amyloid burden or cognition between patients with SSRI treatment compared to untreated patients regardless of a previous depression.

So far, only two studies have evaluated the effects of SSRI-treatment on amyloid burden using amyloid-PET showing controversial findings. Brendel et al. (2018) showed no significant reduction of amyloid deposition in ^18^F-Florbetapir-PET between SSRI-treated and untreated MCI and AD patients with a prior history of depression (Brendel et al., 2018). Results are in line with our findings, as we did not detect significant differences in amyloid burden between SSRI-treated and untreated patients in a comparable patient cohort. In addition, we did not find any significant differences in MCI and AD groups without prior depression nor in cognitively normal patient groups with or without depression. Consistent with our findings, in a previous study by Bartels et al. (2018) CSF Aβ levels were unaffected by SSRI treatment in MCI patients with a history of depression.

In contrast to those findings and to our findings, Cirrito et al. (2011) showed a significant lower cortical binding of the ^11^C-labeled amyloid tracer Pittsburgh Compound-B (^11^C-PiB) in cognitively normal patients that have received fluoxetine, citalopram or escitalopram treatment (mean duration 35 months) compared to patients with no history of SSRI use. The Thioflavin-S derivate ^11^C-PiB was the first amyloid tracer applied in human PET studies with high sensitivity and specificity for the detection of amyloid plaques. Limitations of the ^11^C-labeld tracer due to its availability and short half-life were overcome with the development of ^18^F-labeled amyloid tracers, including ^18^F-Florbetapir. ^18^F-labeled tracers show comparable binding properties and clinical performance to ^11^C-PiB and therefore, differences cannot be mainly explained by the use of different amyloid tracers (Johnson et al., 2013; Barthel and Sabri, 2017; Villemagne et al., 2017). In the study by Cirrito et al. (2011), all participants that took SSRIs had a history of depression while our cohort also included patients that used SSRI due to other reasons than depression which might be the main reason of discrepant results. However, after separating participants with and without history of depression, there were no differences between SSRI+ and SSRI− subjects in our cohort.

We could show that the duration of SSRI treatment correlated inversely with amyloid burden in both, cognitively normal and cognitively impaired patients, consistent with the findings of Cirrito et al. (2011). The time of onset and duration of exposure to SSRIs seems to be a crucial factor as SSRI-treatment might have preventive properties reducing Aβ accumulation. Hypothesizing that SSRIs influence Aβ metabolism promoting the non-amylogenic APP processing pathway leading to a lower amount of soluble neurotoxic Aβ forms and lower amyloid plaque burden, only long-term treatment might contain AD pathologies in a protective way. Furthermore, the development of AD pathologies takes many years and only short-term treatment might not be sufficient in order to reduce Aβ accumulation. This theory is supported by the results of a large Danish population-based study in which patients who received only one prescription of SSRI antidepressants had an increased rate of dementia compared to subjects unexposed to antidepressants, while continued long-term antidepressant treatment (six to nine prescriptions) was associated with a reduced rate of dementia (Kessing et al., 2009). Additional factors that might be modulated by long term SSRI-treatment include depressive symptoms, stress and neuroinflammation with a possible beneficial effect on cognition.

Preclinical studies indicate that the decrease of Abeta levels after SSRI treatment is dose-dependent (Cirrito et al., 2011; Sheline et al., 2014). A dose-dependent decrease of Abeta levels was first demonstrated by Cirrito et al. (2011) as they showed a reduction of ISF Aβ-levels by 12–16% using 5 mg/kg of citalopram while 10 mg/kg reduced Aβ-levels by 24%. However, in our cohort, we could not detect a dose-effect on the cerebral amyloid load in ^18^F-Florbetapir-PET. One possible explanation of theses divergences might be an inter-individual variation in the efficacy of SSRI-treatment. Thus, the dose of SSRI treatment may vary greatly between individual patients and bias a grouped analysis.

It has previously been shown that the ApoE ε4 allele influences the response to different antidepressants in geriatric depression. Murphy et al. (2003) showed that patients carrying the ε4 allele showed a rapid onset of the noradrenergic and specific serotonergic antidepressant mirtazapine action, whereas paroxetine-treated patients with the ε4 allele were slow to respond (Murphy et al., 2003). However, ApoE genotype had no effect on the treatment outcome with SSRIs in the current study. Although, ApoE4 carriers showed significantly higher SUVR compared to non-carriers in cognitively impaired patients as well as in cognitively normal subjects.

Limitations of this study include the retrospective setting. Especially inconsistencies in SSRI-treatment protocols (doses, type of SSRI, duration of treatment) have to be considered as an important limitation. Furthermore, more detailed information on the number and extent of depressive episodes were not available in the dataset. In addition, inconsistencies between the groups regarding the history of depression might have led to a possible bias. Another limitation is the limited number of patients in some of the studied sub-groups.

Overall, the effect of SSRI-treatment on AD-pathologies in humans remains controversial. While preclinical studies and first clinical data showed promising results, a beneficial effect neither on amyloid load nor cognition could be confirmed so far.

## Conclusion

The effect of SSRI-treatment on AD-pathologies remains unclear. However, preclinical data and the negative correlation between the time of SSRI treatment and amyloid load continue to suggest a possible beneficial effect of SRRI-treatment on the pathogenesis of AD. A controlled randomized prospective study on the effect of SSRIs on AD-pathologies is necessary in order to overcome limitations of previous studies evaluating treatment effects in a dose- and time-controlled manner. Furthermore, more information of the mechanism of action of SSRI-treatment are needed, including secondary and tertiary preventive properties, e.g., possible modulation of depressive symptoms and stress of SSRIs in the development of AD.

## Data Availability Statement

The raw data supporting the conclusions of this article will be made available by the authors, without undue reservation.

## Ethics Statement

All ADNI participants provided informed written consent, which was approved by each site’s Institutional Review Board. The patients/participants provided their written informed consent to participate in this study.

## Author Contributions

CB and YB designed the project, analyzed the data, and wrote the manuscript. Both authors contributed to revising the manuscript and approved the final version.

## Conflict of Interest

The authors declare that the research was conducted in the absence of any commercial or financial relationships that could be construed as a potential conflict of interest.

## Publisher’s Note

All claims expressed in this article are solely those of the authors and do not necessarily represent those of their affiliated organizations, or those of the publisher, the editors and the reviewers. Any product that may be evaluated in this article, or claim that may be made by its manufacturer, is not guaranteed or endorsed by the publisher.

## Footnotes

1
http://adni.loni.usc.edu

2
https://adni.loni.usc.edu/study-design/
